# Performance evaluation of a new high-sensitivity time-of-flight clinical PET/CT system

**DOI:** 10.1186/s40658-018-0229-4

**Published:** 2018-12-01

**Authors:** Li Huo, Nan Li, Heyu Wu, Wenjia Zhu, Haiqun Xing, Jiantao Ba, Tong Wang, Fang Li, Hui Zhang

**Affiliations:** 1 Department of Nuclear Medicine, Peking Union Medical College Hospital, Beijing, China; 2 SinoUnion (Beijing) Healthcare Technologies Co., Ltd, Beijing, China; 3 Department of Biomedical Engineering, Tsinghua University, Beijing, China

**Keywords:** PoleStar m660 PET/CT, Performance, Time-of-flight PET/CT, NEMA standard

## Abstract

**Background:**

PoleStar m660 is a newly developed clinical PET/CT system with time-of-flight (TOF) capability. The aim of this study is to characterize the performance of the new system. Spatial resolution, sensitivity, scatter fraction, and noise equivalent count rate (NECR) were measured on the scanner according to the NEMA NU 2-2012 protocol. The timing resolution was measured using a rotating line source that orbited around the center of field of view (CFOV) at a radius of 20 cm. The image quality phantom was also imaged to quantify the percent contrast, percent background variability, and residual error. The impacts of data acquisition time and bed overlap on the PET image quality were also evaluated using phantom and patient studies.

**Results:**

The transverse (axial) spatial resolutions were 3.59 mm (3.67), 4.08 mm (4.65), and 5.32 mm (6.48) full width at half maximum (FWHM) at 1 cm, 10 cm, and 20 cm, respectively, off the CFOV. The measured sensitivity was 10.7 cps/kBq at the CFOV and 10.4 cps/kBq at 10 cm off the CFOV. The peak NECR was 216.7 kcps at an activity concentration of 29.1 kBq/ml, and the scatter fraction was 38.2%. An average of 435 ps FWHM timing resolution was measured. For the image quality phantom, the contrast recovery ratios ranged from 33.9 to 76.4%, while the background variability ranged from 4.7 to 2.0%. In the preliminary clinical study, no noticeable difference in the image quality was observed when the scan time for the whole body and brain was reduced to 1 min/bed and 3 min, respectively. The tested 21% bed overlap showed no significant difference in the image quality compared with the default 38% bed overlap setting.

**Conclusions:**

The physical performances of the PoleStar m660 PET/CT system showed good sensitivity, count rate performance, and timing resolution. The improved performance could help to reduce the acquisition time and bed overlap in the clinical application without detectable compromise in the image quality.

## Background

Time-of-flight (TOF) positron emission tomography (PET) imaging has long been recognized for potential improvement in image quality and/or reduction in image acquisition time [1–6]. With the advent of fast scintillators such as lutetium oxyorthosilicate (LSO) and lutetium-yttrium-orthosilicate (LYSO), as well as the advances in fast coincidence electronics, TOF-PET imaging has become widely available in current commercial clinical PET/CT scanners [7–12]. TOF-PET leads to a relative signal-to-noise ratio (SNR) gain inversely proportional to the system coincidence timing resolution [13]. Thus, the improvement in the timing resolution has always been the main focus in TOF-PET systems. Traditionally, photomultiplier tubes (PMTs) with high quantum efficiency, fast rise time, and low transit-time spread are used in TOF-PET, and system coincidence timing resolution of around 500 ps full width at half maximum (FWHM) can be achieved [7–10]. The recent development of silicon photomultipliers (SiPMs) has led to improve the timing resolution around or below 400 ps FWHM in some newly available systems [14, 15]. A newly announced SiPM-based commercial system has reported a system timing resolution of less than 300 ps [16]. Although SiPMs are promising for improved timing resolution, high-performance PMT-based PET detectors with optimized design can also achieve a significant improvement in timing resolution compared with the existing commercial systems [12, 17]. PoleStar m660 (SinoUnion Healthcare Inc., Beijing, China) is a newly introduced PMT-based high-performance PET/CT scanner, and an initial evaluation of a prototype system has been reported by Huo et al. [18] showing a system timing resolution of 434 ps FWHM. The purpose of this work is to evaluate the physical performance of a newly installed commercial PoleStar m660 PET/CT scanner in the Peking Union Medical College Hospital (PUMCH) using the National Electrical Manufacturers Association (NEMA) protocol NU-2 2012 [19]. Furthermore, phantom studies and initial clinical studies were conducted to assess image quality with respect to the acquisition time as well as the acquisition bed overlaps.

## Methods

### Scanner

A PoleStar m660 PET/CT scanner was recently installed at PUMCH that consisted of a high-speed 64-slice helical CT subsystem and a PMT-based whole-body TOF-PET subsystem. The main technical features of the CT and PET components were summarized in Tables 1 and 2, respectively. The PET subsystem has 24 detector buckets arranged in a cylindrical geometry. Each detector bucket has two detector modules along the trans-axial direction, and along the axial direction, there are either three detector modules for the standard configuration or four detector modules for the mView configuration. The scanner under evaluation has an mView configuration. The detector module uses a block detector design [18]. Each detector module contains a 14 × 14 LYSO scintillator array with a crystal size of 3.73 mm × 3.73 mm × 20 mm and coupled to four PMTs through light sharing. The detector ring diameter was 84 cm, and the patient bore size was 70 cm. The system average energy resolution was 12.0% ± 0.5% FWHM, and the energy window of the system was set to 425–650 keV. The coincidence timing window was set at 4.1 ns, and the TOF information was acquired and encoded with a resolution of 15.625 ps. During histogramming, the listmode data was rebinned into 13 TOF bins with a bin width of 296.875 ps. The PET scanner acquired data in three-dimensional (3D) configuration, and the singles rate on each crystal was recorded to estimate random coincidences of the measured data [20]. For the whole-body scan, by default, a 38% bed overlap (45 out of 117 planes) was set for all the data acquisitions; however, the bed overlap can be user-adjusted to satisfy different clinical needs.

**Table 1:** PoleStar m660: major CT technical characteristics

Detector material	Solid State Ceramics
Number of detector rows	32
Number of detectors per row	864
*Z*-axis coverage	32 × 0.625 mm
Tube anode heat capacity	8 MHu
Maximum generator power	80 kW
Tube voltages	80/100/120/140 kV
Tube current	10–667 mA
Scan mode	Topogram, helical, axial
Maximum number of CT slices	64
Spiral scan time	100 s

**Table 2:** PoleStar m660: major PET technical characteristics

Detector material	LYSO
Crystal size	3.73 mm × 3.73 mm × 20 mm
Crystals per block	196
PMTs per block	4
Number of detector blocks	144/192 (mView configuration)
Axial field of view	166 mm/223 mm (mView configuration)
Detector ring diameter	840 mm
Coincidence window width	4.1 ns
Scan mode	Static, dynamic, gating
Number of image planes	87/117 (mView configuration)
Plane spacing	1.87 mm

### Scanner performance measurement

The physical performances were investigated at the site of clinical installation of the system following the procedure outlined by the NEMA NU 2-2012 protocol [19] when applicable.

#### TOF timing resolution

To facilitate the measurement, a rotating jig provided by the manufacturer (SinoUnion Healthcare Inc., Beijing, China) was used, with a 3-mm-diameter Ge-68 line source of 11.1 MBq. The line source was rotated around the center of the scanner parallel to the axial direction of the scanner at a radius of 20 cm. No attenuation or scatter correction was assumed in the study, and the dead time loss was also ignored due to the low activity of the source. The differences in the arrival times for all the coincidence photons passed through the scanner center of field of view (CFOV) were recorded, and timing histograms were generated for all the possible lines of response (LORs). In theory, two symmetrical peaks in the histograms could be observed with a distance corresponding to the flight time across the 40 cm diameter. Gaussian fitting was applied to the two peaks in the timing histogram, and the average FWHM was reported as the system TOF timing resolution.

#### Spatial resolution

Spatial resolution measurements were performed using three point sources inside a thin glass capillary tube (1 mm in diameter). The point sources were prepared by dropping F-18 FDG solution of 185 MBq/ml through the capillaries. The drops were carefully handled to keep the axial length of the drops around 1 mm. In the transverse FOV, the three point sources were positioned at 1 cm, 10 cm, and 20 cm along a horizontal line. Data were acquired at the axial center and three eighths of the axial FOV separately. About 1.6 million coincidence counts were collected at each position to satisfy the sufficient statistical analysis, which were then sorted into 3D sinograms. Two methods were employed in reconstructing the point sources. First, the 3D sinogram was subtracted with random sinogram and then rebinned into 2D sinogram by FORE method [21]. Point source images were then reconstructed by 2D filtered back projection (FBP) using an unapodized ramp filter with a cutoff at the Nyquist frequency. Second, maximum likelihood expectation maximization (MLEM) reconstruction with the inclusion of point spread function (PSF) was used, and random correction was directly applied in the iterative process. Five iterations were used in the reconstruction. The image matrices for both reconstructions were 512 × 512, with the pixel size of 1.88 mm × 1.88 mm. The attenuation and scatter corrections were ignored in both methods, and no post-smoothing filter was applied. FWHMs of the point source response functions were calculated by linear interpolation between adjacent pixels in radial, tangential, and axial directions.

#### Sensitivity

A 700-mm-long line source filled with 10 MBq F-18 FDG solution in a plastic tube was placed in the center of the trans-axial FOV parallel to the axial direction of the scanner. Successive acquisitions were performed by surrounding the line source with five concentric aluminum sleeves progressively. The scanning duration was 60 s for each acquisition. Same measurements were repeated at 10 cm above the center of trans-axial FOV.

The acquired data were sorted and rebinned into 2D sinograms and applied with decay and/or random correction. Five data sets were acquired and fitted to derive an exponential relation between the true count rate for each sleeve and sleeve thickness. The count rate with no absorption is extrapolated to a zero-thickness sleeve to calculate the system sensitivity. Furthermore, the axial sensitivity profile was calculated as a function of axial offset, in proportion to the true counts measured for each slice. Same data analysis was repeated for the sensitivity measurement acquired at 10 cm off the center of the scanner.

#### Scatter fraction and noise equivalent count rate

A 700-mm-long solid scatter cylinder with an outside diameter of 200 mm was used. A line source filled with 921.3 MBq F-18 FDG solution was inserted axially into the cylinder at a 45-mm radial offset below the phantom center. The scatter phantom was positioned at the center of FOV, in parallel with the scanner axis. A total of 38 emission frames were acquired for more than 15 h, with a 600-s acquisition time and a 900-s delay for each frame.

The emission data for each frame were sorted and rebinned into 2D prompt sinogram by single-slice rebinning method. Singles rates were recorded frame-by-frame as well for random estimation and correction. Both prompt and random sinograms were masked within a 24-cm-diameter FOV in the center of the scanner. All the voxels outside the masked region were set to zero. Then, each prompt projection was shifted in a radial direction to align the pixels with the maximum value in the center of the sinogram. Under a 40-mm-wide strip centered on the peak, the scatter sinogram could be estimated by linearly interpolating the scattered counts under the peak in the strip region and adding all the counts outside the strip. Then, the scatter fraction and noise equivalent count rate (NECR), as a function of activity level, were determined.

#### Image quality

The start activity of F-18 FDG solution filled in the torso phantom was 128 MBq to ensure a background concentration of 5.5 kBq/ml in the scan. Four small spheres were filled with radioactive solution eight times to the background to simulate hot lesions, and the two large spheres were filled with water as cold lesions. The torso phantom was centered in the FOV of the scanner, and the spheres were aligned to the center of the axial FOV. To simulate the background activity from the body outside the FOV, a 70-cm NEMA scatter phantom with a line source was positioned adjacent to the torso phantom. The acquisition time was 242 s to simulate a whole-body scan of 100 cm axial imaging distance in 30 min.

Data was sorted into 3D sinogram and corrected for random, normalization, dead time loss, attenuation, and scatter. The torso phantom images were reconstructed using 3D ordered subset expectation maximization (OSEM) algorithm. In order to evaluate the influence of PSF and TOF on image quality performance, four iterative OSEM reconstruction algorithms were investigated, shown as non-TOF, non-TOF-PSF, TOF, and TOF-PSF. Reconstruction settings were the same as set in the clinical scan protocols routinely used at PUMCH. The subset used in all the reconstructions was 10, with iterations of 4 for non-TOF and non-TOF-PSF and 2 for the other two TOF reconstruction algorithms. Gaussian filtering of 4.0 mm FWHM was applied to all the reconstructed images. The image matrix was 192 × 192, with a pixel size of 3.15 mm × 3.15 mm.

For the analysis, circular regions of interest (ROIs) were drawn on the six spheres in the reconstructed images as well as 60 background regions of the same size as the spheres in different image slices. As described in the NEMA NU 2-2012 protocol, for each sized sphere, the contrast recovery coefficient (CRC) was computed as the ratio of the measured average sphere-to-background ROI count ratio and the actual sphere-to-background activity concentration ratio, and the background variability was calculated as the ratio of the ROI count standard deviation (SD) of the same sized background ROIs and the average background ROI counts. In addition, a ROI of 3 cm in diameter was drawn (in each slice of the phantom) in the central cylindrical insert. The residual error was calculated as the ratio of the average counts in the lung insert ROI to 60 background ROIs.

#### Clinical imaging performance

In order to evaluate the routine clinical imaging performance of the scanner, preliminary patient studies were conducted. The use of the patient data was approved by the Ethics Committee at PUMCH.

#### Scan time

In this study, the influence of scan time per bed position on the PET image quality was explored. A whole-body scan as well as a brain scan was performed on a patient weighing 75 kg (body mass index (BMI) of 30.8) to visually compare the image quality under different scan time. An injection of 417 MBq (11.3 mCi) ^18^F-FDG was administered to the patient 60 min prior to the scan. During the whole-body scan, the PET data were acquired for 3 min per bed position with a total of five bed positions (38% bed overlap). In order to compare the image qualities under different scan time, the acquired listmode data were divided and sorted into five groups of sinogram, corresponding to a scan time of 15 s, 30 s, 1 min, 2 min, and 3 min per bed position, respectively. The images were reconstructed using the OSEM-TOF algorithm with two iterations and ten subsets, 192 × 192 image matrix with 3.15 mm × 3.15 mm pixel size, and filtered by a 4.0-mm FWHM Gaussian filter. During the brain scan, the PET data were acquired for 10 min (one bed position) and divided into three groups of sinograms corresponding to a scan time of 3 min, 5 min, and 10 min, respectively. Images were reconstructed using the OSEM-TOF algorithm, with four iterations and ten subsets, 512 × 512 image matrix with 1.16 mm × 1.16 mm pixel size, and filtered by a 2.5-mm FWHM Gaussian filter.

To further evaluate the quantitation impact of the reduced scan time, 11 patients were enrolled in the study and performed whole-body scans using the same acquisition conditions and data processing methods as described above. For each patient, five sets of PET images were generated which correspond to a scan time of 15 s, 30 s, 1 min, 2 min, and 3 min per bed position, respectively. For data analysis, a spherical volume of interest (VOI) with 3 cm diameter was placed in the liver for all the patients, and mean standardized uptake values (SUVmean) and maximum SUVs (SUVmax) were calculated and analyzed.

#### Bed overlap

To explore the effect of bed overlaps on the PET image qualities, both phantom and preliminary patient studies were carried out in this study. A uniform Ge-68 cylinder phantom with an activity of 13 MBq (0.35 mCi) was scanned using the clinical whole-body protocol for two bed positions with 3 min per bed position. The axial center of the cylinder phantom was placed in the overlapped region between the two bed positions, and the data were acquired using discretely changed bed overlaps of 5, 10, 15, 20, 25, 30, 35, 40, and 45 planes of a total of 117 planes, respectively. Images of the cylinder phantom were reconstructed using the clinical whole-body reconstruction settings as described in the above section. For data analysis, a cylinder ROI (10 cm in diameter and 6 cm in length) was placed in the center of the reconstructed images to axially cover the overlapped region. The corresponding mean and maximum value of the reconstructed activity concentration in the ROI were calculated to investigate the impact of bed overlaps.

In the preliminary patient study, acquisitions with 21% bed overlaps (25 planes) and 38% bed overlaps (45 planes) were first performed on a patient to visually compare the image qualities. The patient was injected with 423 MBq (11.4 mCi) of ^18^F-FDG. Whole-body scans were conducted first for five bed positions with 21% bed overlap at 50 min post-injection and then for six bed positions with 38% bed overlap at 75 min post-injection. For each bed position, a 3-min acquisition was performed. Images were reconstructed using the clinical whole-body reconstruction settings as described above and visually compared for the two bed overlap settings. For quantitation analysis, 12 patients were enrolled in the study and performed two successive whole-body scans with 21% bed overlaps and 38% bed overlaps respectively using the same protocol as described above. SUVmean and SUVmax were calculated for spherical VOIs with 3 cm diameter placed in the liver for all the patients.

## Results

### Timing resolution

**Fig. 1:**
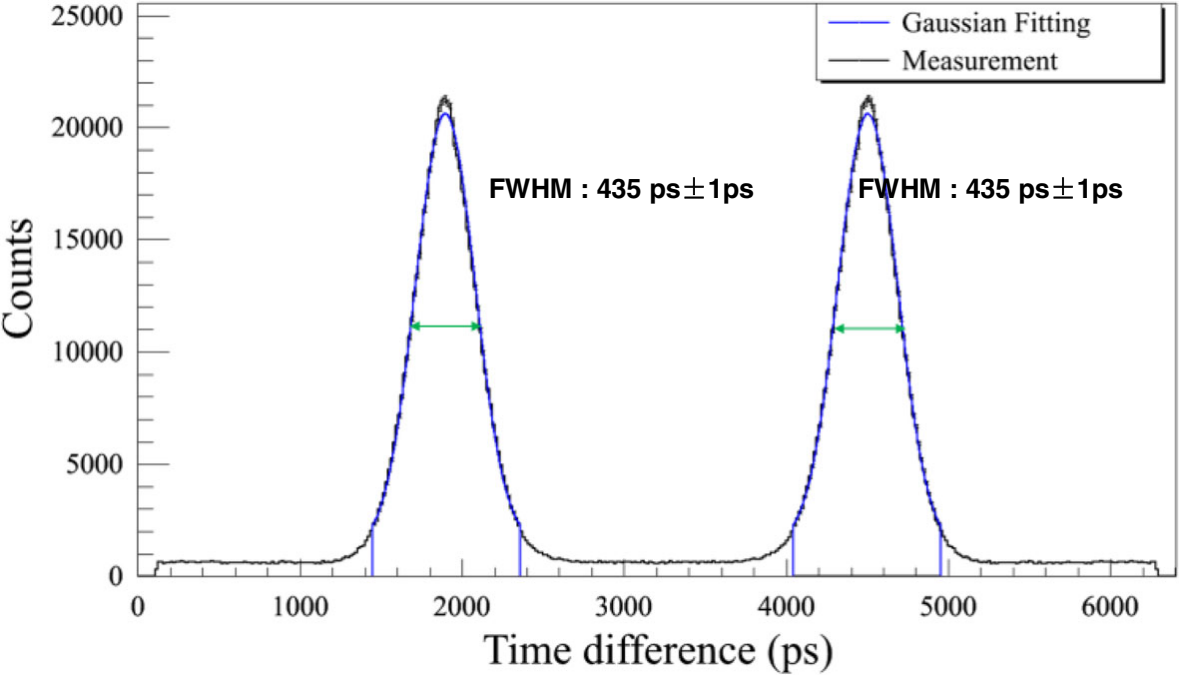
The true timing spectrum acquired using a rotating line source that orbits around the CFOV at a radius of 20 cm. Gaussian fitting was applied to the two peaks, and the average FWHM was reported as the timing resolution of the system

The coincidence timing spectra fitted by Gaussian function were shown in Fig. 1. The timing resolutions derived from both symmetrical peaks were the same, and the average of coincidence timing resolution was 435 ps ± 1 ps.

### Spatial resolution

The reported spatial resolutions using FBP reconstruction in average over both transverse and axial positions were 3.63 mm, 4.27 mm, and 5.7 mm at 1 cm, 10 cm, and 20 cm, respectively, from the center of the FOV (Table 3). Considering the depth of interaction effects in the crystals and more accurate geometric model than the line-integral model in FBP, the MLEM reconstruction method with PSF option was used, and average spatial resolutions of 1.55 mm, 2.01 mm, and 2.08 mm were measured at a corresponding radial offset respectively (Table 3).

**Table 3:** NEMA NU 2-2012 performance characteristics

				FWHM
Spatial resolution (mm)	FBP	1 cm	Transverse	3.59
			Axial	3.67
		10 cm	Transverse radial	4.38
			Transverse tangential	3.78
			Axial	4.65
		20 cm	Transverse radial	5.95
			Transverse tangential	4.68
			Axial	6.48
	PSF-OSEM	1 cm	Transverse	1.41
			Axial	1.68
		10 cm	Transverse radial	1.67
			Transverse tangential	2.07
			Axial	2.3
		20 cm	Transverse radial	1.81
			Transverse tangential	2.11
			Axial	2.32
Sensitivity (cps/kBq)		0 cm		10.7
		10 cm		10.4
Scatter fraction				38.2%
Peak NECR				216.7 kcps at 29.1 kBq/ml
Peak trues				818.6 kcps at 38.2 kBq/ml

### Sensitivity

**Fig. 2:**
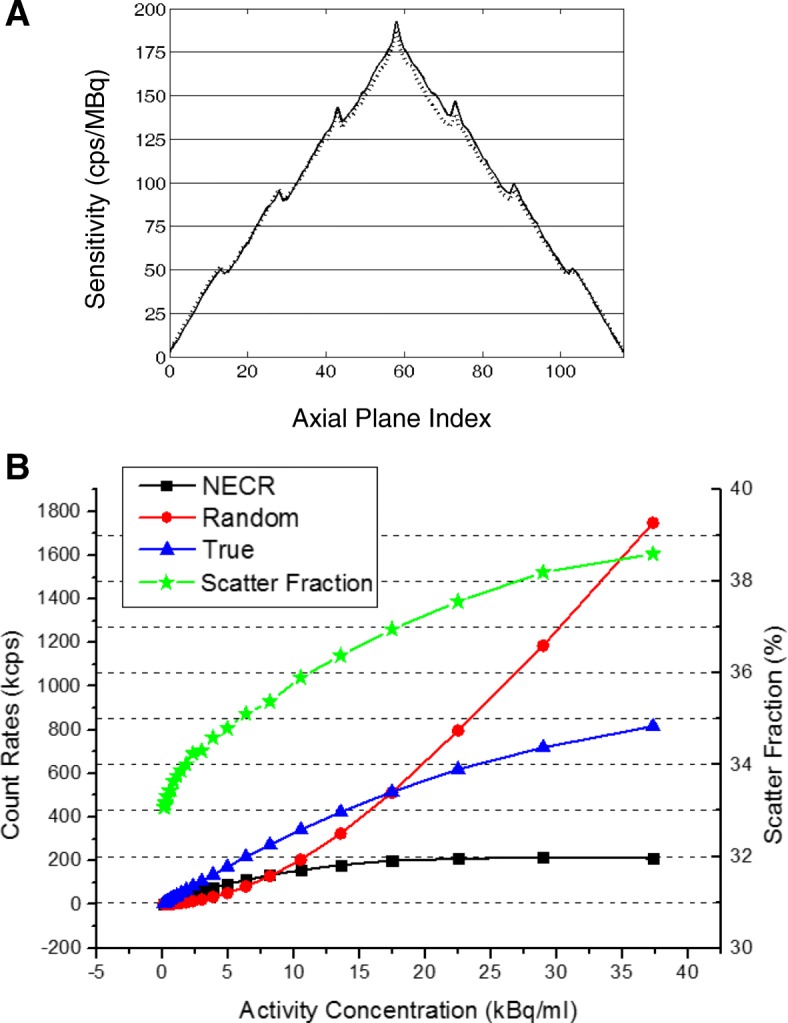
**a** Plots of axial sensitivity at the CFOV (solid line) and at a 10-cm offset radially from the CFOV (dotted line). **b** Plot of NECR (square), randoms (circle), true (triangle), and scatter fraction (star) as a function of activity concentration

In the sensitivity measurement, the activity of the line source was expected to be low enough to ignore the random coincidences and dead time loss [19]. The system sensitivity at the CFOV of the scanner was measured to be 10.7 cps/kBq. As the line source moved 10 mm away from the scanner’s center axis, the sensitivity was measured to be 10.4 cps/kBq. The axial sensitivity profiles for both radial positions were shown in Fig. 2.

### Scatter fraction and NECR

As reported in Table 3, the scatter fraction at peak NECR was measured to be 38.2%, at the default energy window of 425–650 keV. The scatter fraction as a function of different activity concentration was plotted in Fig. 2 as well, which showed a relatively stable increase as the activity concentration increases.

Figure 2 also plotted the random, true, and NECR curves as a function of activity concentrations. For the smoothed random correction (1R), the peak NECR was 216.7 kcps at a concentration of 29.1 kBq/ml, and the maximum true count rate was 818.6 kcps at a concentration of 38.2 kBq/ml, as listed in Table 3.

### Image quality

**Fig. 3:**
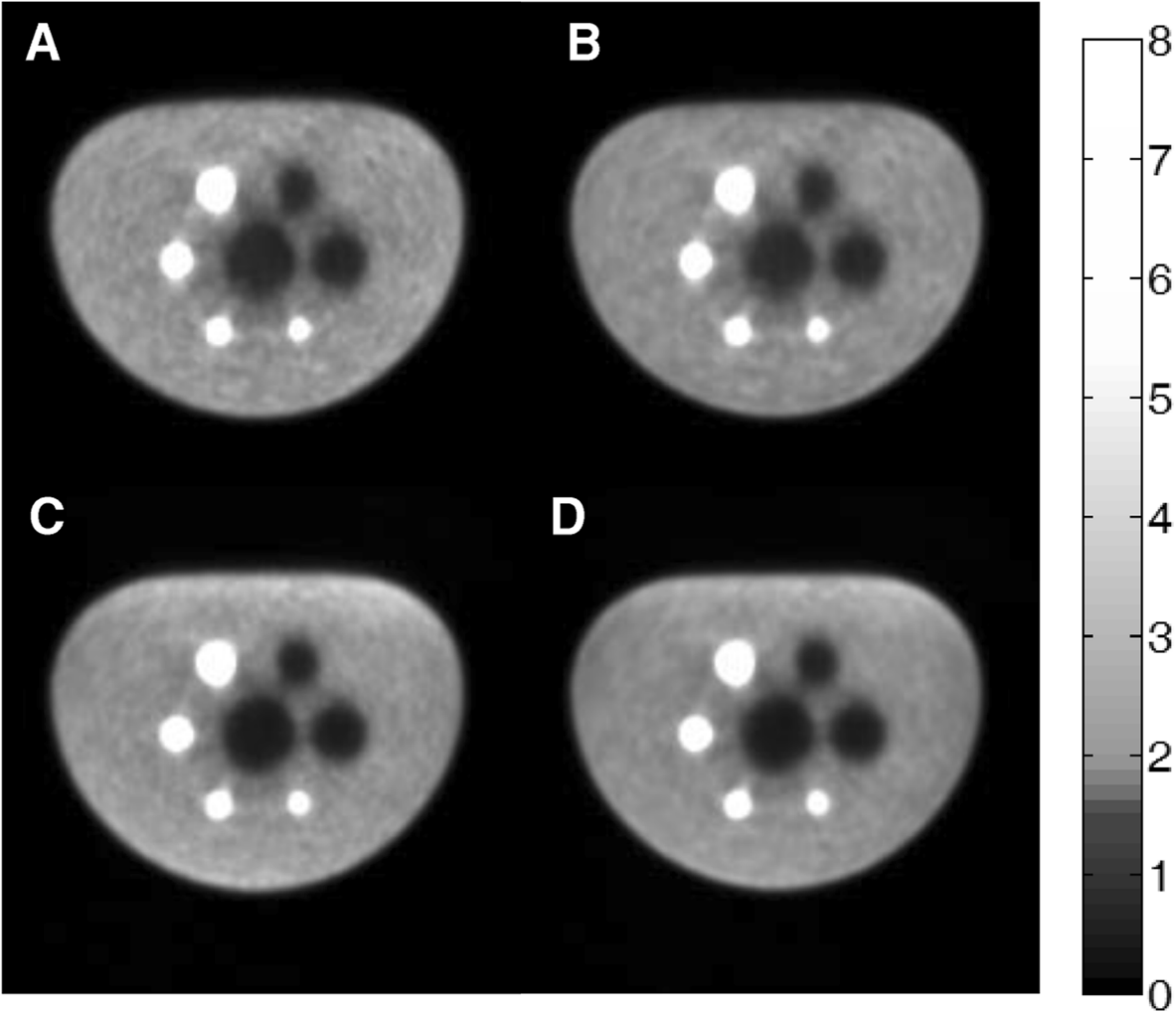
2D cross-sections of reconstructed image quality phantom at the axial center of the spheres with a 192 × 192 image matrix. The ratios of the activity concentrations within hot rods, background, and cold rods were 8:1:0. **a** 3D-OSEM. **b** 3D-OSEM with PSF function. **c** 3D-OSEM with TOF information. **d** 3D-OSEM with both TOF information and PSF function

The percent recovery coefficients and background variability values for the sphere-to-background ratios of 8:1 were listed in Table 4. It is worthwhile to note that for Table 4, the reconstruction settings were the same as set in clinical applications. Central slices of the NEMA torso phantom images for all the four reconstructions were shown in Fig. 3. Compared with non-TOF reconstructions, the TOF reconstructions increased both the hot and cold contrasts at similar background. However, the TOF reconstruction had more evident benefits in cold contrast recovery, with much more clear and round boundary around the cold spheres and the lung region.

**Table 4:** Contrast, variability, and average lung residual for 8:1 sphere-to-background ratio

Sphere Size (mm)	Contrast (%)				Background variability (%)			
	Non-TOF	Non-TOF-PSF	TOF	TOF-PSF	Non-TOF	Non-TOF-PSF	TOF	TOF-PSF
10	34.3	33.9	41.0	38.2	4.0	4.1	4.6	4.7
13	47.4	48.2	54.2	53.0	3.7	3.8	4.4	4.6
17	60.2	61.5	65.8	64.9	3.4	3.4	4.3	4.4
22	70.3	72.0	74.4	74.4	2.9	3.0	4.3	4.4
28	65.6	62.7	73.0	71.0	2.4	2.6	4.2	4.4
37	70.2	67.6	76.4	74.7	2.0	2.3	4.4	4.6
Lung residual (%)	26.4	27.8	14.9	15.4

Figure 3 also showed that the PSF led to the improvement in contrast for the small spheres (especially the 10-mm-diameter sphere) with a sharper boundary. However, the amelioration of contrast became subtle in larger spheres.

The average of residual errors for different reconstruction algorithm was also listed in Table 4. As previously indicated, TOF reconstructions could improve cold contrast by about 15% and therefore exhibited significant smaller residual error compared with non-TOF reconstructions.

### Clinical imaging performance

#### Scan time

**Fig. 4:**
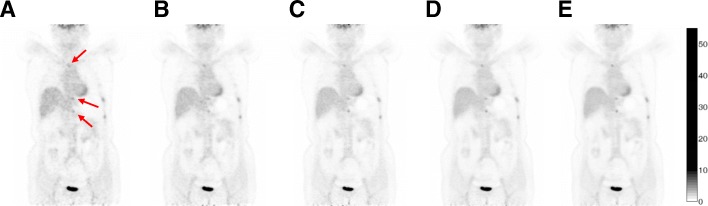
Coronal slices of clinical PET whole-body images acquired with the PoleStar m660 PET/CT. In the whole-body scan, the emission data were acquired for a total of five bed positions with different acquisition time: 15 s per bed (**a**), 30 s per bed (**b**), 1 min per bed (**c**), 2 min per bed (**d**), and 3 min per bed (**e**)

**Fig. 5:**
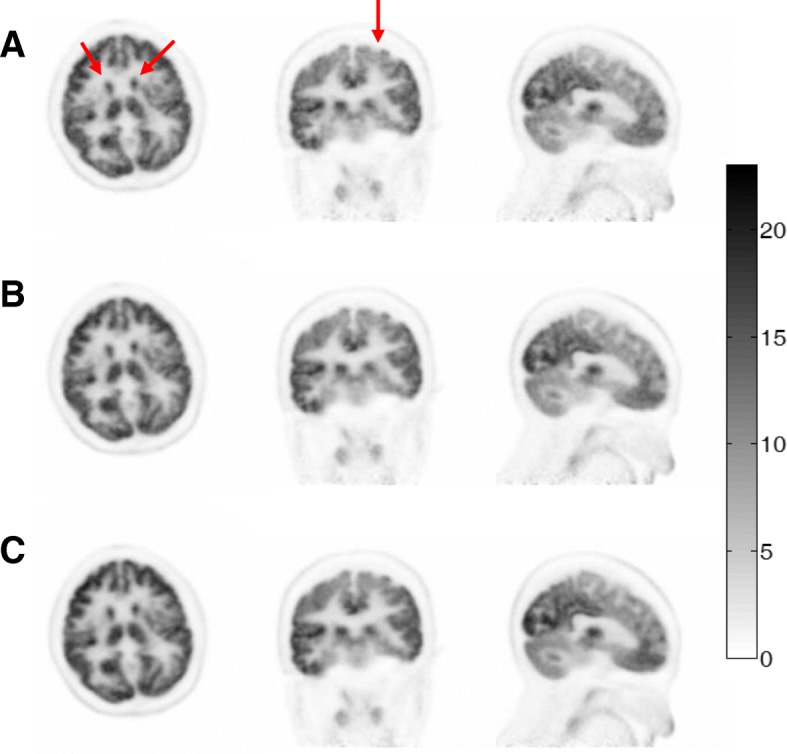
Representative slices of the clinical PET brain images acquired with the PoleStar m660 PET/CT. The single-bed reconstructions were compared with different acquisition time: 3 min (**a**), 5 min (**b**), and 10 min (**c**)

Figure 4 showed representative coronal slices of the whole-body PET images reconstructed with 15 s, 30 s, 1 min, 2 min, and 3 min per bed position acquisition, respectively. Although the image quality degrades as the acquisition time per bed position decreases, the suspected tumors could still be distinguished in all the images for the whole-body scan, even down to 15 s per bed acquisition time, as indicated by the arrows in the image. Visual inspections of the images suggested that satisfactory image quality could be achieved using 1-min acquisition per bed position for the whole-body scan. Figure 5 showed representative PET images of the brain scan reconstructed with 10 min, 5 min, and 3 min data acquisition, respectively. Compared with the 10-min brain scan, images reconstructed with 3-min acquisition time could retain almost all the visible gross features such as the gyri and sulci of the cerebrum, with a little increased noise. And visual details of glucose metabolism in the brain could be clearly identified in all the cases.

**Fig. 6:**
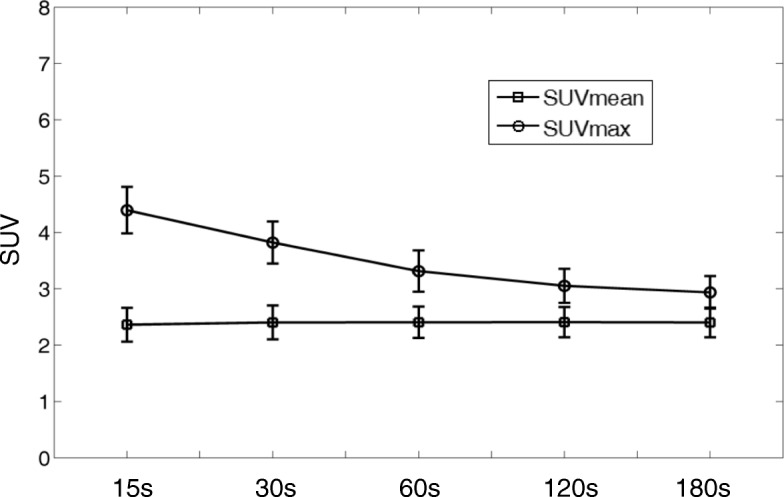
Means and standard deviations of SUVmean and SUVmax as a function of acquisition time for 11 patients

Figure 6 plotted the mean values and the standard deviations of the SUVmean and SUVmax in the liver ROIs in 11 patients under different scan times. As shown in Fig. 6, the noise in the reconstructed images increases with reduced scan time, leading to a larger variation in SUVmax, especially when the scan time is less than 2 min per bed position. For SUVmean, however, the average process in ROI could reduce the effect of noise, and thus, a much stable value as well as deviation was observed.

#### Bed overlap

**Fig. 7:**
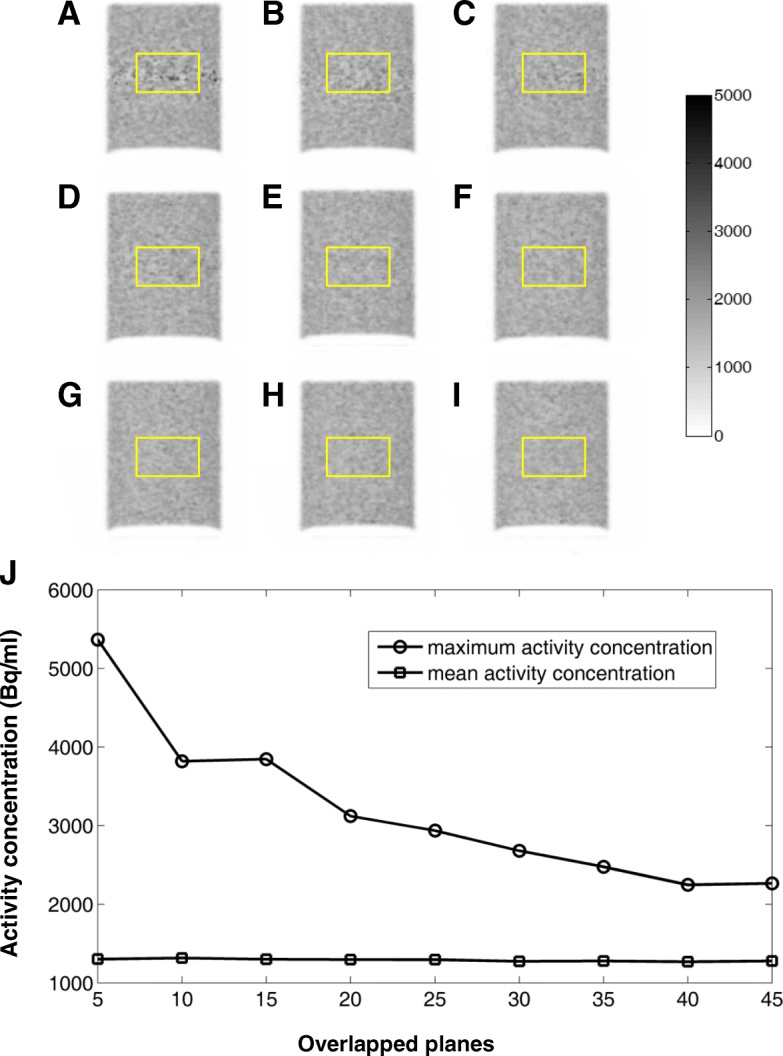
Coronal slices of the images from a Ge cylinder scan with different PET overlaps in the unit of planes: 5 (**a**), 10 (**b**), 15 (**c**), 20 (**d**), 25 (**e**), 30 (**f**), 35 (**g**), 40 (**h**), and 45 (**i**). The emission data were acquired for a total of two bed positions. **j** Maximum and mean value of activity concentration as a function of overlapped planes

Figure 7 showed representative coronal slices of the uniform Ge-68 cylinder phantom scanned with different bed overlaps. Visual inspection suggests that there was no significant artifact in the reconstructed images for the bed overlaps down to 25 planes, while for smaller bed overlaps, strip artifacts started to appear. Quantitative analysis results in the central ROI that across the overlapped acquisition region for different bed overlaps were also shown in Fig. 7. Mean activity concentration values in the ROI were much stable, with less than 3% relative change for all the bed overlaps. For the maximum values in the ROI, however, a large variation was observed. For the 25 planes bed overlap case, a relative increase of 29.5% in the maximum value was found compared with the default 45 planes bed overlap case.

**Fig. 8:**
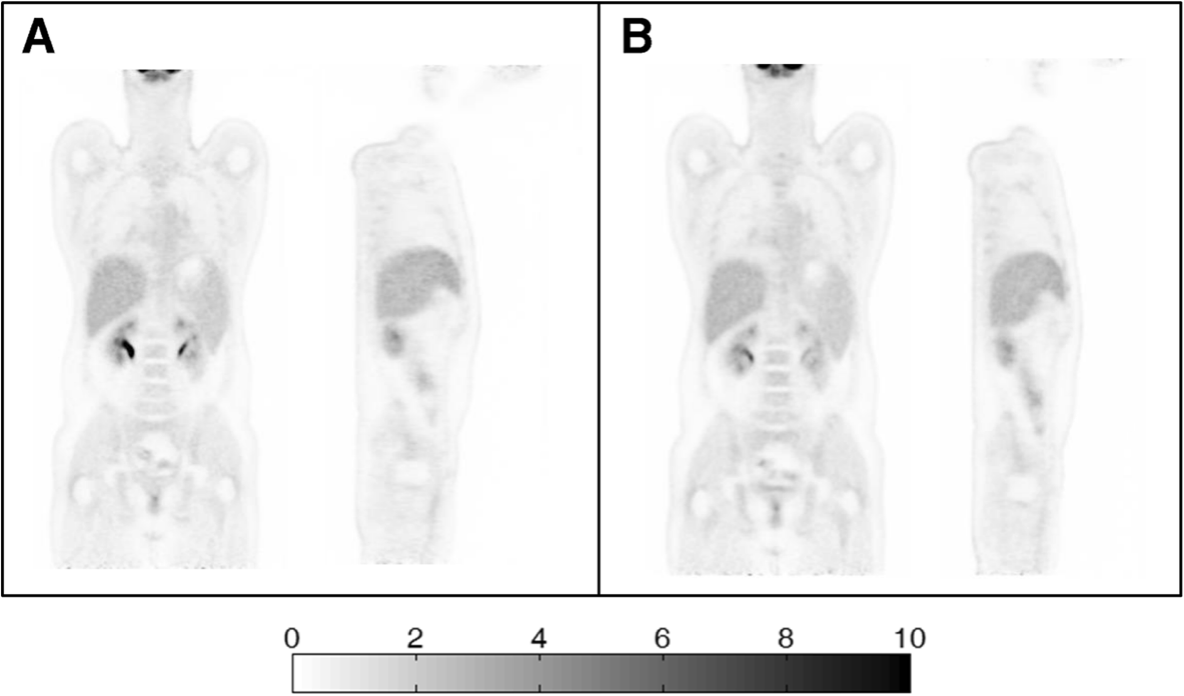
Reconstructed coronal and sagittal images for a patient scan. The emission data were acquired for five beds with an overlap of 25 planes (**a**) and for six beds with an overlap of 45 planes (**b**). Both reconstructions offered similar image quality without any discontinuous artifacts along the axial direction

**Fig. 9:**
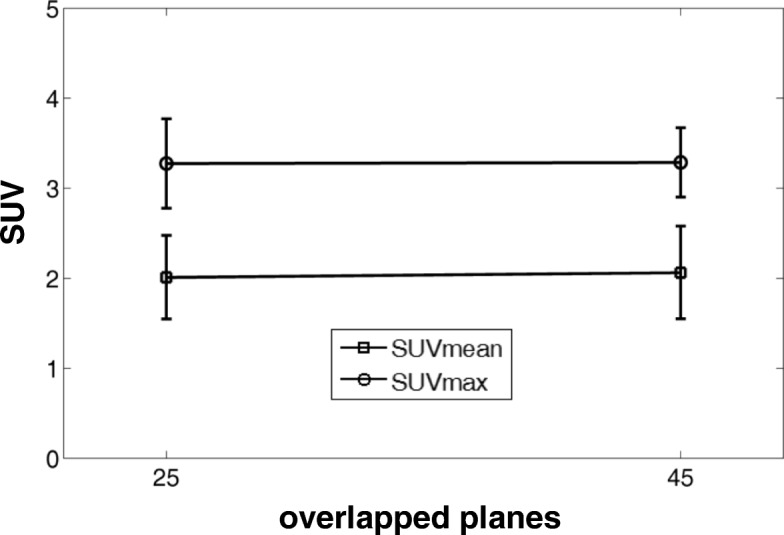
Means and standard deviations of SUVmean and SUVmax calculated from overlap settings of 25 and 45

Figure 8 showed the whole-body scan images with 25 and 45 planes bed overlap, respectively. Compared with the default 45 planes bed overlap, the reduction of the bed overlap to 25 planes did not introduce any obvious artifact along the axial direction. Figure 9 showed the quantitative analysis results in the reconstructed liver ROI images of the 12 patients with the overlap settings of 25 and 45 planes, respectively. For both SUVmax and SUVmean, no significant difference in the standard uptake values was found in the study between the two overlap settings. However, compared with the default overlap of 45 planes, the reduction to the overlap of 25 planes led to the increased noise and a larger deviation in SUVmax, as shown in Fig. 9.

## Discussion

In this study, we followed the NEMA NU 2-2012 protocol to evaluate the performance of the PoleStar m660. The evaluation results showed that the spatial resolution of PoleStar m660 was equivalent to or better than that of the state-of-the-art commercial PET/CT scanners in the market [7–12]. In addition, the inclusion of PSF generated a more uniform spatial resolution distribution throughout the FOV. The measured high sensitivity and NECR of the scanner indicated its capability of acquiring considerable photon counts in a wide range of activity concentration, leading to noise-robust reconstructed images in clinical cases. This was demonstrated in Fig. 5, where the visual details of the glucose metabolism in the brain could still be identified even when the scan time was reduced to 3 min.

The system coincidence timing resolution measurement is not specified in the NEMA NU 2-2012 standard. Previous studies reported using Ge-68 or FDG line sources suspended axially in the radial center of the scanner FOV, parallel to the axial direction of the scanner [10]. In this study, we have used a rotating Ge-68 line source to measure the timing resolution of the scanner. The coincidence timing spectrum, as shown in Fig. 1, exhibits two peaks, and the distance between the peaks corresponds to the flight time across the rotating diameter. System coincidence timing resolution was calculated as the average of the FWHM of the two peaks. In this way, a 435-ps timing resolution was measured for the scanner, which, to our knowledge, is the best timing resolution that was ever reported with commercially available PMT-base PET systems. As was discussed in [13], the gain of the imaging SNR when TOF information is used in the image reconstruction is proportional to the object size and inversely proportional to the timing resolution. This was demonstrated in the patient study in Fig. 4. Normally, patients of big BMI lead to increased attenuation and reduced detected photons, resulting in degradations in the image quality. However, Fig. 4 showed that for the patient with a BMI of 30.8, even with 1-min acquisition per bed position, satisfactory image quality could be achieved, and in addition, the reduction of the scan time did not significantly compromise the visual detection of lesions.

**Fig. 10:**
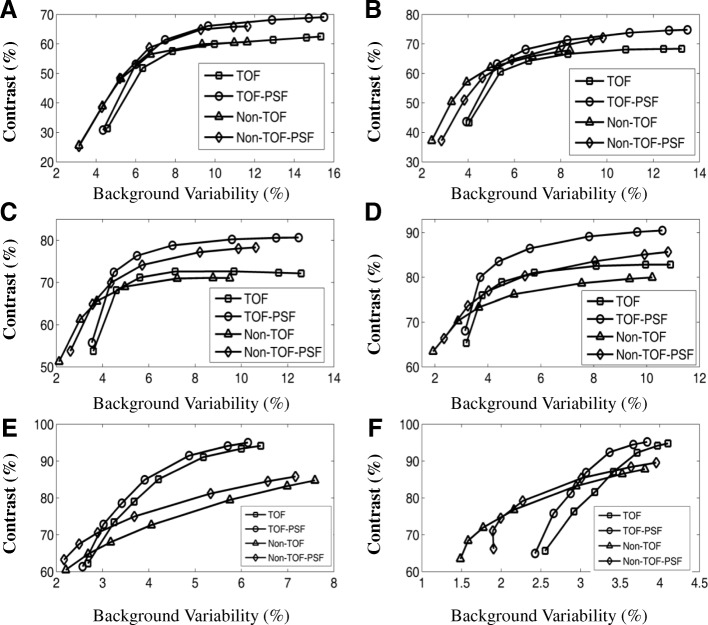
Contrast vs background variability for different reconstruction algorithms (TOF, TOF+PSF, non-TOF, and non-TOF+PSF) and a number of iterations (points of each graph correspond to 1, 2, 3, 5, 10, 15, and 18 iterations). These plots are used to estimate the performance balance for hot rods with an inner diameter of 10 mm (**a**), 13 mm (**b**), 17 mm (**c**), and 22 mm (**d**) and cold rods with an inner diameter of 28 mm (**e**) and 37 mm (**f**)

An objective comparison of the image quality between the non-TOF and TOF reconstructions using the NEMA torso phantom was listed in Table 4. Improvements in the contrast recovery were evident from non-TOF reconstructions to TOF reconstructions, especially for large size cold spheres; however, the background variabilities also increased with TOF reconstructions. It is worthwhile to note that the results in Table 4 were obtained with the same reconstruction settings as in the clinical whole-body protocols, where four iterations were used for non-TOF reconstructions and two iterations were used for TOF reconstructions. Clinical reconstruction settings such as those used at PUMCH were chosen largely based on the compromise between the reconstruction speed and the visual image quality. The visual inspection of image quality, however, depends on personal preferences, which suggests that the clinical settings may not necessarily be the most optimal case in terms of objective evaluations. This was demonstrated in Fig. 10 where for different sized spheres in the NEMA torso phantom, we plotted the convergence behavior of the contrast and background variability in terms of iteration numbers for the non-TOF, non-TOF-PSF, TOF, and TOF-PSF reconstructions, respectively. The subset used in all the reconstructions was 10, and the image matrix was 192 × 192 with no smoothing filter applied on the reconstructed images. TOF reconstructions were converged at around 3–5 iterations, while more iterations (> 10) were required for the non-TOF reconstructions, suggesting that two iterations for TOF and four iterations for non-TOF reconstructions in the clinical settings were sub-optimal in terms of objective evaluation. However, with less iteration numbers, the reconstruction will consume much less time, which is preferred in clinical applications.

The limited axial FOV of PET scanner requires multiple bed acquisition to cover the full axial extent of a patient in the whole-body scan. Consecutive bed position is overlapped by 38% (45 overlapped planes of a total of 117 planes) by default in the PoleStar m660 scanner in order to make system sensitivity along the axial direction to be uniform. A reduction in the overlap will inevitably cause non-uniformity in the axial system sensitivity; however, in practice, the impacts on the image quality are subject to evaluation. In both phantom and patient scans in this study, we found no significant degradation in image quality with smaller bed overlaps down to 21% (25 overlapped planes of a total of 117 planes). Quantitative analysis of both SUVmax and SUVmean demonstrated no significant difference in the standard uptake values between the 45 and 25 overlap settings. However, the reproducibility of SUVmax quantification is limited owing to the variability induced by the substantial noise level. The reduction of bed overlaps from 45 planes to 25 planes could lead to one bed position reduction for the same scan range in a typical whole-body scan, and thus lead to improved throughput in routine clinical practices.

## Conclusions

The performance of the PoleStar m660 PET/CT scanner was characterized according to the NEMA NU 2-2012 protocol. The spatial resolution of 3.99 mm (transverse) and 3.67 mm (axial) at 1 cm off the CFOV, average system sensitivity of 10.55 cps/kBq, and peak NECR of 216.7 kcps were observed. Furthermore, the scanner measured a timing resolution of 435 ps, which leads to much improvement in the image quality using TOF reconstruction. The improved performance could help to reduce the scan time and bed overlap in clinical scans, with insignificant degradation in the image quality.

## Abbreviations

3D: Three-dimensional
BMI: Body mass index
CFOV: Center of field of view
CRC: Contrast recovery coefficient
CT: Computed tomography
FBP: Filtered back projection
FDG: Fluorodeoxyglucose
FORE: Fourier rebinning
FWHM: Full width at half maximum
LOR: Line of response
LSO: Lutetium oxyorthosilicate
LYSO: Lutetium-yttrium-orthosilicate
MLEM: Maximum likelihood expectation maximization
NECR: Noise equivalent count rate
NEMA: National Electrical Manufacturers Association
OSEM: Ordered subset expectation maximization
PET: Positron emission tomography
PMT: Photomultiplier tube
PSF: Point spread function
PUMCH: Peking Union Medical College Hospital
ROI: Region of interest
SiPM: Silicon photomultiplier
SNR: Signal-to-noise ratio
SUV: Standard uptake value
TOF: Time-of-flight
VOI: Volume of interest
